# Validity and Reliability of the Cardiac Rehabilitation Barriers Scale in the Czech Republic (CRBS-CZE): Determination of Key Barriers in East-Central Europe

**DOI:** 10.3390/ijerph182413113

**Published:** 2021-12-12

**Authors:** Petr Winnige, Katerina Filakova, Jakub Hnatiak, Filip Dosbaba, Otakar Bocek, Garyfallia Pepera, Jannis Papathanasiou, Ladislav Batalik, Sherry L. Grace

**Affiliations:** 1 Department of Public Health, Faculty of Medicine, Masaryk University, 62500 Brno, Czech Republic; winnige.petr@fnbrno.cz; 2 Department of Rehabilitation, University Hospital Brno, 62500 Brno, Czech Republic; filakova.katerina@fnbrno.cz (K.F.); hnatiak.jakub@fnbrno.cz (J.H.); dosbaba.filip@fnbrno.cz (F.D.); 3 Faculty of Medicine, Masaryk University, 62500 Brno, Czech Republic; 4 Department of Internal Cardiology Medicine, University Hospital Brno, 62500 Brno, Czech Republic; bocek.otakar@fnbrno.cz; 5 Physiotherapy Department, Faculty of Health Sciences, University of Thessaly, 35100 Lamia, Greece; gpepera@uth.gr; 6 Department of Medical Imaging, Allergology & Physiotherapy, Faculty of Dental Medicine, Medical University of Plovdiv, 4002 Plovdiv, Bulgaria; giannipap@yahoo.co.uk; 7 Department of Kinesitherapy, Faculty of Public Health, Medical University of Sofia, 1431 Sofia, Bulgaria; 8 Faculty of Health, York University & KITE-Toronto Rehabilitation Institute, University Health Network, University of Toronto, Toronto, ON M3J 1P3, Canada; sgrace@yorku.ca

**Keywords:** barriers, utilization, cardiac rehabilitation, coronary artery disease, Czech Republic

## Abstract

Cardiovascular rehabilitation (CR) is an effective secondary preventive model of care. However, the use of CR is insufficient, and the reasons for this are not well-characterized in East-Central Europe. This prospective observational study psychometrically validated the recently translated Cardiac Rehabilitation Barriers Scale for the Czech language (CRBS-CZE) and identified the main CR barriers. Consecutive cardiac in/out-patients were approached from January 2020 for 18 months, of whom 186 (89.9%) consented. In addition to sociodemographic characteristics, participants completed the 21-item CRBS-CZE (response options 1–5, with higher scores representing greater barriers), and their CR utilization was tracked. Forty-five (24.2%) participants enrolled in CR, of whom 42 completed the CRBS a second time thereafter. Factor analysis revealed four factors, consistent with other CRBS translations. Internal reliability was acceptable for all but one factor (Cronbach’s alpha range = 0.44–0.77). Mean total barrier scores were significantly higher in non-enrollers (*p* < 0.001), decreased from first and second administration in these enrollers (*p* < 0.001), and were lower in CR completers (*p* < 0.001), supporting criterion validity. There were also significant differences in barrier scores by education, geography, tobacco use, among other variables, further supporting validity. The biggest barriers to enrolment were distance, work responsibilities, lack of time, transportation problems, and comorbidities; and the greatest barriers to adherence were distance and travel. Several items were considered irrelevant at first and second administration. Other barriers included wearing a mask during the COVID-19 pandemic. The study demonstrated sufficient validity and reliability of CRBS-CZE, which supports its use in future research.

## 1. Introduction

Cardiovascular diseases (CVD) are among the leading causes of morbidity and mortality worldwide, including in Central European countries [1]. They account for most hospitalizations, are a frequent cause of incapacity to work, and disability in the Czech Republic [2]. In 2018, according to the Czech Statistical Office, CZK 28.5 billion was spent on CVD treatment, which comprised the greatest proportion of medical expenditures (13% of total) [3].

Secondary prevention can mitigate this burden [4]. Indeed, according to the World Health Organization, 75% of recurrent cardiovascular events can be prevented [4]. Cardiovascular rehabilitation (CR) is an effective secondary preventive model of care that reduces mortality, and positively affects the quality of life of patients with CVD [5,6]. CR offers physical training, education focused on risk factor management, lifestyle and its modification, nutritional therapy, psychological support, and optimized pharmacotherapy [6,7].

Despite all the benefits [5,6] and cost-effectiveness [8], the use of CR is insufficient. Worldwide, 15–30% of eligible patients access CR [9]. Utilization rates are higher in high-income countries (40–50%), where healthcare is readily available [10]. CR is insufficiently available and thus not often practiced in low and middle-income countries [11]. In the Czech Republic specifically, while data are lacking, it is estimated approximately 10–20% of patients access outpatient CR. Another issue is adherence to the rehabilitation program once patients enroll. Data suggest that patients complete an average of 72% of prescribed training sessions [12]. Lower adherence has been demonstrated in the elderly [13], patients with comorbidities, the unemployed, and the less educated [14].

CR barriers occur at multiple levels, including lack of patient and healthcare provider awareness, lack of availability and integration of rehabilitation into the cardiac care system, lack of financial remuneration, and suitable timing of session delivery [15]. Patients report that their main barriers to CR participation are work and family responsibilities, financial costs, exercise concerns, lack of motivation, or long distance from CR facilities [10]. The patient groups that are under-represented in CR often have more barriers, such as for example women citing a lack of social support and the presence of comorbidities such as arthritis, osteoporosis, or urinary incontinence [16]. Most data on CR barriers ironically stem from high-income settings, despite access barriers being greater in low and middle-income countries [11].

To better characterize CR barriers in the Czech Republic, recently we rigorously translated and cross-culturally adapted the CR Barriers Scale (CRBS) [17,18]. The objective of this study was to psychometrically validate the CRBS-CZE with regard to factor structure, reliability and validity. A second aim was to identify the key CR barriers in the Czech Republic, as this has not yet been established. With this knowledge, we may implement appropriate strategies to improve patient participation in CR programs.

## 2. Materials and Methods

### 2.1. Design

This was a prospective, observational study. This study is registered at the Australian New Zealand Clinical Trial Registry with registration number: ACTRN12619001181190, and a published protocol is available elsewhere [17].

### 2.2. Setting

The study took place at University Hospital Brno (UHB), Czech Republic. Around 1000 patients are hospitalized at its Internal Cardiology Clinic every year, and the facility also has an outpatient clinic and CR program.

There is virtually no CR waiting list; the program starts approximately 4–12 weeks after the patient’s discharge, depending on their preference and clinical condition. Note the program was closed due to COVID-19 for approximately three months during the period of study, and there were some restrictions thereafter. It is mandatory to have public health insurance in the Czech Republic, and the health insurance companies fully cover the CR program costs. Patients travel to the program via car or public transportation (most often by bus) in a ratio of 50/50.

The CR program is comprehensive, and is delivered by physiotherapists and physicians. The recommended patients must undergo a cardiopulmonary exercise test before starting the program. Participants are offered three training sessions per week for two months (a total of 24 sessions) [19,20]. Patient education is delivered with each session, as per the guidelines [21].

### 2.3. Procedure

From January 2020, eligible patients at the Internal Cardiology Clinic of UHB (hospital, standard ward, coronary unit) were recruited for 18 months. All patients were informed about the CR program and the study. A questionnaire assessing sociodemographic characteristics and the CRBS-CZE were provided to agreeing participants. If necessary, patients were provided with assistance during completion of the questionnaire.

All data were obtained through patient self-report (or via interview with a physiotherapist if they participant needed assistance), direct measurement, or from medical records. Information on enrolment, adherence, and completion of the CR program was extracted from the CR records two months later.

After three weeks in CR, participants were asked to complete the CRBS-CZE for a second time. This was to test whether scores are responsive to change and also to establish the psychometrics are sound in assessment of CR adherence barriers as well as enrolment.

### 2.4. Participants

Inpatients and outpatients who had not been referred to CR were approached. Eligible patients had to meet the following criteria: ≥18 years and diagnosis of coronary heart disease, specifically coronary artery disease (CAD), acute coronary syndrome, heart failure, arrhythmias, or valve defects, and/or having undergone percutaneous coronary intervention or coronary artery bypass graft surgery, as per CR indications in clinical practice guidelines [22]. Exclusion criteria included significant acute cardiovascular risk, implanted cardioverter-defibrillator/pacemaker, orthopedic/neurological disorders severely limiting movement training, and participation-excluding mental disorders (e.g., schizophrenia, advanced dementia). For factor analysis, 10 participants (at least 5) per variable must be obtained [23], corresponding to 105–210 participants.

### 2.5. Measures

The assessed socio-demographic characteristics of participants included age, sex, marital status, highest education attained, income, and employment parameters (e.g., entrepreneurship, shift work, physical demands), all assessed using investigator-generated items. The clinical characteristics included diagnosis, risk factors (anthropometry, blood pressure, dyslipidemia, diabetes), lifestyle (degree of physical activity, duration of medium-high-intensity activity/week, tobacco use, alcohol consumption, number of standard drinks), and psychosocial well-being (stress, visual analog scale, 1–10; depression/anxiety, diagnosed and self-report [yes/no]). The travel time to the CR facility was also self-reported by the participants. Since selected characteristics can influence CR participation, some of the variables were used to test the construct validity of CRBS-CZE.

The CR utilization data were used to assess criterion validity. Patients attending at least one training session by 12 weeks post-discharge were considered enrollers. Adherence was defined as the proportion of the 24 prescribed sessions attended. Patients attending > 75% of total training sessions were considered completers.

The validated CRBS assesses barriers affecting CR enrolment and/or adherence at the level of patients and their experience in the health system [18]. The English version contains 21 items (barriers) related to four subscales: healthcare system factors, logistical barriers, work/time conflicts, and comorbidities/functional status. Items are rated on a five-point Likert scale (1—most minor barrier, 5—most significant barrier); a higher score denotes greater barriers to CR enrolment or adherence. At the end of the CRBS is an open-ended item (22) where patients may add other factors that limit participation.

The CZE version contains revisions of some items and examples for the Czech context. For each item, a not applicable option is available. If participants completed > 80% of the items, the average score was calculated.

### 2.6. Analysis

Software Statistica (TIBCO Software Inc., Palo Alto, CA, USA) and SPSS v28 (IBM, New York, NY, USA) was used for analyses. The level of statistical significance was set at *p* < 0.05.

First, we considered participant characteristics, as well as mean CRBS scores and proportion of items rated not applicable using descriptive statistics. Next, we assessed the CRBS-CZE factor structure through principal component factor analysis with varimax rotation. Factors with eigenvalues > 1 were considered, and the scree plot was also examined to determine number of factors. Factor loading values > 0.2 were interpreted to determine item loadings. Cronbach’s alpha was used to evaluate the internal reliability of CRBS-CZE overall and for each identified factor. Scores above 0.70 are generally considered acceptable [24].

Comparison of total and subscale barriers scores based on utilization indicators was undertaken using independent samples *t*-tests, and Pearson correlation for adherence. To test construct validity, we tested for differences in overall CRBS-CZE scores by the patients’ socio-demographic and clinical characteristics, again using the two tests listed above as applicable.

Differences in CRBS total and scores between first and second administration were assessed using paired sample *t*-tests in enrollees. Also differences in scores at the second administration in completers and non-completers were tested.

Finally, mean items and subscale scores were perused to identify the top barriers in this context, at both administrations. Any additional barriers reported by the respondents were content analyzed by P.W. and L.B. After reviewing all the responses and mapping out general categories, this involved systematically categorizing each response manually, and then counting frequency of the codes.

## 3. Results

**Figure 1:**
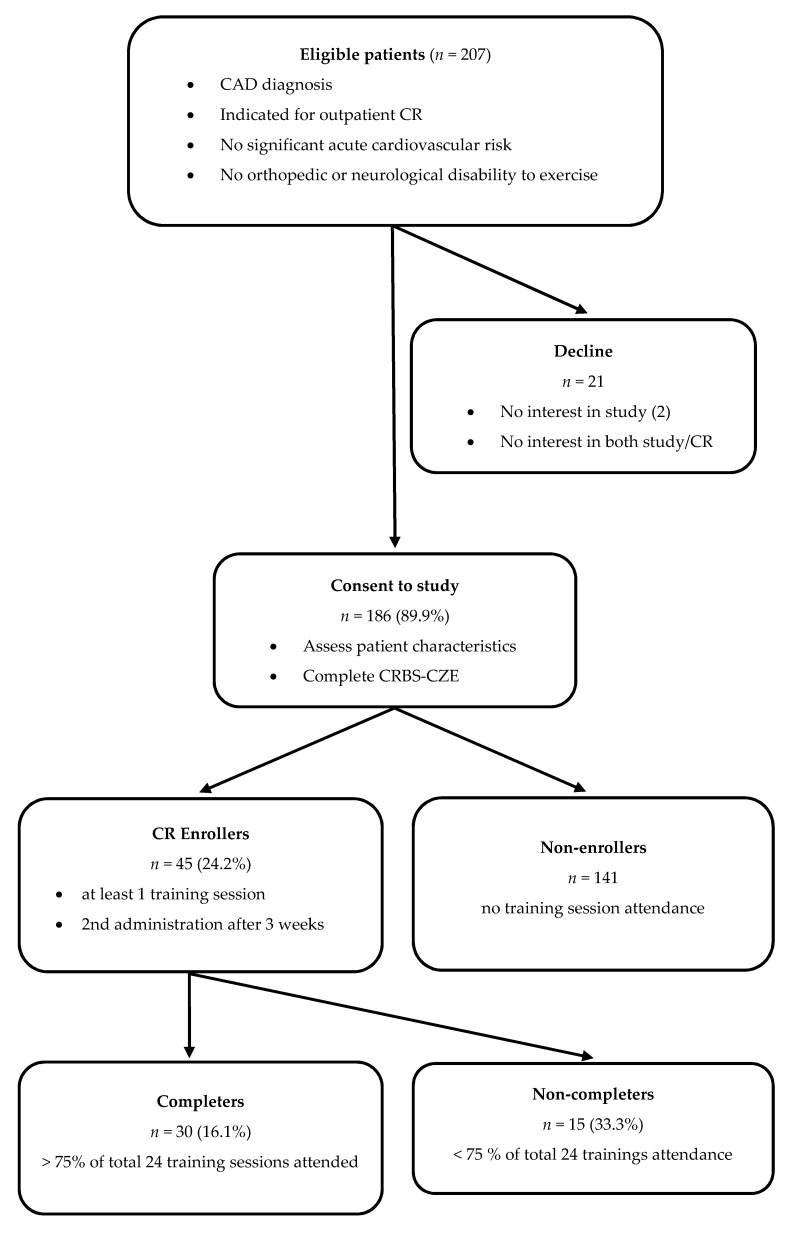
Flowchart of the study.

Figure 1 shows participant flow. Table 1 shows the participant characteristics. The 45 (24.2%) CR enrollees completed on average 70.8 ± 29.8% of the 24 prescribed sessions.

### 3.1. Factor Structure and Reliability

CRBS item completion rates are shown in Table 2. Three items in particular were not highly relevant in this context at the first administration, namely severe weather, provider informing them CR is not necessary, and not being contacted by the CR program subsequent to referral. At the second administration, there were two which were not considered relevant by more than half of respondents: many people with heart problems don’t go (and they are fine) and it took too long to get referred into the program.

Table 3 shows the results of the factor analysis from first administration. As shown, four factors were extracted. Internal reliability of the overall scale was good (Cronbach’s alpha = 0.77); for subscales it ranged from 0.44–0.72.

### 3.2. Criterion and Construct Validity

Initial CRBS-CZE scores were compared by utilization to evaluate criterion validity. As shown at the bottom of in Table 1, total CRBS score were related to CR enrolment, adherence, and completion.

Higher barriers were also reported by individuals living in rural environments, with lower levels of education and income. Barriers were also related to clinical characteristics such as post-myocardial infarction condition, risk factors including dyslipidemia, family history of CAD, tobacco use, or harmful use of alcohol (overall shown in Table 1, and by subscale in Table 4). No other differences were observed.

### 3.3. Repeat Administration of the CRBS-CZE

In addition to the first completion during CR recruitment, enrollers were asked to complete the CRBS-CZE three weeks after enrollment for the second time. Characteristics of the 42 participants doing so are shown in the Table S1. Enrollers were found to have significantly lower barriers in the second than in the first administration. This is shown in Table 5, including by subscale; a significant reduction was also observed for each participant.

The Table S1 also shows the characteristics of CR completers and non-completers who filled out the CRBS a second time. As also shown in Table 5, CR completers had significantly lower barrier scores at the second administration as well (total and all subscales), again further bolstering criterion validity of the CRBS-CZE. There were significant decreases in barrier items 1, 3–5, 11–13, 15, 17, 19–21; there were no significant increases in any barriers (Table 2).

### 3.4. Greatest Barriers

Table 2 shows the mean item scores. At the first administration pre-CR, the distance from the CR facility was rated as the most significant barrier to enrolment, with the average one-way transport time per CR session being 50 min. Other significant barriers (average value > 2/5) included lack of time, work responsibilities, comorbidities, or transportation problems.

Additional barriers to enrolment that were reported open-ended were morning exercise session time (*n* = 2), frequent check-ups with their doctor (*n* = 2), needing to exercise with a mask/respirator due to COVID-19 (*n* = 2), exercise fear (*n* = 1), inflexible training session time (*n* = 1), and parking problems (*n* = 1).

At the second administration during CR, travel was rated as the most significant barrier to program adherence (Table 2). Other significant barriers included distance, work responsibilities, and comorbidities. Additional barriers reported open-ended were deterioration of health (*n* = 1), planned cardiac spa stay (*n* = 1), and summer holidays (*n* = 1).

## 4. Discussion

Despite robust evidence of the benefits of CR, even of each additional session attended [25], utilization remains low [9]. Patient’s barriers to CR utilization should be evaluated, hence the emergence of the CRBS and its 15 translations [18,26,27,28,29,30,31]. In the Czech Republic, data on barriers and patient participation in CR are lacking.

Through this study, the psychometric validity and reliability of the Czech version of the CRBS were established. Results are consistent with other versions of the CRBS, identifying four subscales [18,26,29,31]. However, in this translation, healthcare system factors hung together with comorbidities, rather than perceived need; this may explain the low internal reliability of the latter subscale, which is a limitation of this translation. Even the perceived need subscale internal reliability was of borderline acceptability, whereas in other translations Cronbach’s alpha ranged from 0.56–0.89 across the subscales. Those using this scale in future should bear this in mind when analyzing subscale data.

Sociodemographic and clinical characteristics shown to be related to CR barriers were generally consistent in this study [18,26,28,30], supporting CRBS-CZE validity. However, no significant difference was found between the sexes. Previous research has shown this, but that the nature of men and women’s barriers differ (i.e., which items are rated highest), which would likely apply herein as well [32]. Novel in this context was the assessment of harmful use of alcohol; rates are somewhat higher in our region [33], and thus we must consider how this may impact our patient’s participation in our CR programs. For example, patients may miss sessions due to veisalgia or levels of intoxication.

The other study aim was to identify key CR barriers in our region, as these do differ significantly by context [18,26,27,28,29,30,31]. Distance from the CR facility was rated as the most significant barrier. This was expected because in the Czech Republic, as in the rest of Europe, CR is delivered mainly at a center [34], and is currently offered only in three large cities [35]. The average transportation time to get to each session was 50 min one-way, despite that drive times of no longer than 30 min are recommended [36]. This was compounded by other significant barriers around transportation. CR remains sub-optimally available in the Czech Republic, and home-based offerings would certainly circumvent this, and the other main barriers around work and other time constraints, as discussed below [35].

A novel aspect of this study was the repeat administration of the CRBS, other than test-retest reliability. In addition to the first completion during recruitment, CRBS-CZE was also completed three weeks after enrollment. Analysis revealed significantly lower perceived barriers, across every subscale. This likely reflects diminishing concern about exercise training, patient’s mastery of the components of CR with experience, and the support provided by the team. It also likely reflects self-efficacy in overcoming some of their logistical barriers, realizing that CR is applicable to patients of all ages, and gaining more energy as they became fit. Clearly barriers to enrolment and adherence differ, such that it would be important to discuss with patients over their continuum of care, to support them in optimally engaging in CR.

Another novel aspect of this study was the reporting of items which patients perceived as not applicable. Some barriers were not relevant; for example, all patients in the cohort were referred and almost all were contacted by the CR program to enroll as a function of the study. Additionally, the weather is fairly temperate in the region, with mild winters and thus weather was not a relevant barrier. Moreover, unfortunately physicians do not routinely refer and encourage their patients to attend CR, but only do so with patients who have the opportunity to interact with physiotherapists. Given the power of a physician’s recommendation [37], this must change (and hence the item should not be removed from the scale). Interestingly, once in CR, the barrier regarding the perception of other patients that do not attend was no longer perceived as relevant, likely due to their experience of observing similar patients engaging in CR. Results highlight the importance of getting patients to that first appointment, so that we can engage them and allow for many of their barriers to be naturally overcome.

Other barriers raised were in some instances already assessed in the items, but others were novel. One in particular related to the COVID-19 pandemic and is discussed in more detail below. However, in relation to time constraints, patients reported that the time of day of the sessions or inflexibility in terms of scheduling were barriers. Kinesophobia was also raised [38]. It may be warranted to undertake further research to update the CRBS (i.e., CRBS-II) to ensure that irrelevant items are removed (as considered above) and unassessed barriers are added, while ensuring an applicability to a broad range of contexts, and maintaining the open-ended item to capture additional barriers that a patient may experience.

These are the first documented rates of CR utilization in the Czech Republic. As the present study revealed, the utilization of CR in this area is low. Of eligible patients approached, 19/21 declined because they were not interested in CR (the other two declined only the research, due to the time-consuming nature of data collection). The CR enrolment rate among consenting patients was 24.2%, with CR-enrolling patients attending approximately 70% of the 24 prescribed sessions, but only 16.1% ultimately completing at least 75% of those. While the mean barrier scores among enrollees were much lower, clearly the barriers are paramount. These rates are consistent with previous research reporting a participation rate of approximately 10–20% in various jurisdictions around the globe [39].

There are several implications. Given the results of this study, which identified mainly logistical and work/time barriers, one possible solution is to improve the availability of home-based programs [40,41]. Home-based programs are a safe and effective alternative for low and moderate-risk patients [42,43]. With the advancement of technologies, home-based CR is buttressed, which is also the subject of current research in the Czech Republic [44,45,46]. This is timely given the current global situation with the COVID-19 pandemic; through telerehabilitation, participants can still receive all CR components [47]. Indeed, the European Association of Preventive Cardiology considers telerehabilitation to be relevant for all patients with CVD who cannot visit CR centers regularly, and suggests it should continue after the COVID-19 pandemic [48].

Indeed, the COVID-19 pandemic was declared during the study, which limited the operation of UHB, the center-based CR program, and hence data collection. Thus, the barriers identified may have been somewhat different during this time, but it was our experience that patients who were concerned about COVID-19 did not want to participate in both the study and CR. Nevertheless, this study does provide a first look in the field at CR barriers for cardiac patients during the pandemic. Indeed for “other” barriers, patients reported using a mask during exercise as an additional barrier to participation. This was a measure we had to implement for some time, in accordance with Czech Government and UHB regulations. This additional barrier was raised by only two participants, however.

Caution is warranted in interpreting these results. The limitations of this study include generalizability. Whether results are applicable to cardiac patients outside the city of Brno, or to other countries in the region, requires further study. A minority proportion of patients did not meet study inclusion/exclusion criteria because they were already referred. Some eligible patients declined to participate due to a lack of interest in the study and CR. These patients may have had different barriers than participants consenting to the study. Moreover, results are only generalizable to in or out-patients who have been informed about CR, and who are referred to a center-based CR program.

Second, due to the nature of the objectives, adjusted analyses were not performed; indeed, some barriers may be conflated with patient sociodemographic characteristics (e.g., patients living in rural areas would be more likely to report distance as a major barrier). Finally, due to the nature of the design, causal conclusions cannot be drawn.

## 5. Conclusions

Through this study, we demonstrated that the Czech version of CRBS has sufficient validity and reliability—although it is low for one subscale. In addition, validity of the CRBS was demonstrated across the continuum of CR care for the first time, reinforcing the utility of the CRBS in assessing barriers to both enrolment and adherence. This supports its future use. Indeed, key barriers to CR in the region were identified for the first time: mainly logistical factors and work/time conflicts, which are likely generalizable to the East-Central Europe area. These findings highlight the importance of offering alternative models of CR, such as home-based programs delivered with technology. It is hoped by offering CR in this alternative setting, barriers would be reduced, such that the use of CR, and secondary prevention of CVD would be improved.

## Figures and Tables

**Table 1:** Participant characteristics (*n* = 186).

Characteristics	Mean (SD) or Frequency (%)	Mean Total CRBS Score (SD) by Characteristic †		t/r	*p*
**Sociodemographic**					
Age (mean ± SD)	59.5 ± 8.8	NA		0.02	0.718
Sex (*n*, % female)	45 (24.1)	1.86 (0.42)	1.84 (0.49)	0.25	0.795
Marital status ∥ (*n*, % married)	155 (83.3)	1.89 (0.48)	1.82 (0.42)	0.73	0.462
Educational attainment ∥ (*n*, % > high school)	34 (18.2)	1.69 (0.42)	1.91 (0.48)	2.51	0.012
Work status ∥ (*n*, % employed)	125 (67.2)	1.84 (0.48)	1.88 (0.46)	0.55	0.582
Individual income ∥ (*n*, % > 33,697 CZK/month)	41 (22.0)	1.72 (0.45)	1.90 (0.47)	2.13	0.047
Rurality ∥ (*n*, % > 30 min commute time to CR one-way by usual mode)	109 (58.6)	2.11 (0.44)	1.58 (0.39)	8.44	<0.001
**Clinical**					
Referral indication of CAD ° (*n*, % yes)	77 (41.3)	1.92 (0.53)	1.84 (0.42)	1.17	0.240
Post-acute myocardial infarction (*n*, % yes)	99 (53.2)	1.96 (0.49)	1.78 (1.20)	2.53	0.012
Angina pectoris (*n*, % yes)	22 (11.8)	1.81 (0.46)	1.88 (0.47)	0.63	0.526
Current/previous PCI (*n*, % yes)	159 (85.4)	1.93 (0.48)	1.90 (0.60)	0.20	0.838
Current/previous CABG (*n*, % yes)	6 (3.2)	NR			
Current/previous HF (*n*, % yes)	3 (1.6)	NR			
Current/previous arrhythmia (*n*, % yes)	15 (8.1)	NR			
Current/previous valve issue (*n*, % yes)	6 (3.2)	NR			
**Risk factors**					
BMI (mean ± SD)	29.5 ± 4.6	NA		−0.01	0.860
Waist circumference (mean ± SD)	106 ± 12	NA		−0.02	0.734
Family history of CAD ∥ (*n*, % yes)	119 (63.9)	1.75 (0.39)	2.06 (0.55)	4.36	<0.001
Hypertension ∥ (*n*, % yes)	117 (62.9)	1.83 (0.43)	1.95 (0.53)	1.63	0.104
Dyslipidemia ∥ (*n*, % yes)	112 (60.2)	1.79 (0.45)	1.99 (0.48)	2.92	0.003
Diabetes ∥ (*n*, % yes)	43 (23.1)	1.83 (0.40)	1.88 (0.49)	0.61	0.539
**Heart-healthy behaviors**					
Physical activity ∥ (*n*, % inactive §)	88 (47.3)	1.90 (0.49)	1.84 (0.45)	0.87	0.383
Tobacco use ∥ (*n*, % current)	72 (38.7)	1.97 (0.47)	1.81 (0.47)	2.36	0.019
Use of alcohol ∥ (*n*, % harmful ‡)	32 (17.2)	2.11 (0.52)	1.83 (0.46)	3.10	0.002
**Psychosocial well-being**					
Stress ∥ (VAS, 1–10; mean ± SD)	4.1 ± 2.4	NA		−0.11	0.111
Depression and/or anxiety (*n*, % yes, diag./self-report ∥)	13 (6.9)/21 (11.2)	NR/1.77 (0.44)	NR/1.88 (0.48)	1.05	0.293
**CR utilization**					
Enrollment (*n*, % yes)	45 (24.2)	1.61 (0.46)	1.96 (0.44)	4.65	<0.001
Adherence (mean % of sessions completed ± SD)	70.8 ± 29.8%	NA		−0.51	<0.001
Completion (*n*, % yes)	30 (16.1)	1.41 (0.36)	1.99 (0.45)	6.70	<0.001

SD = standard deviation, CRBS = Cardiac Rehabilitation Barriers Scale, NA = not applicable because continuous, CR = cardiac rehabilitation, CAD = coronary artery disease, PCI = percutaneous coronary intervention, CABG = coronary artery bypass graft, NR = not relevant as cell sizes too small, HF = heart failure, BMI = body mass index, VAS = visual analogue scale; ∥ Presents self-report data. All other data measured or extracted from patient charts; † Category shown in column on farthest left in parentheses presented in left-hand column and opposite shown to right. ° Defined as coronary artery stenosis > 50%, primomanifestation. § Defined as > 150/75 min of moderate/vigorous intensity physical activity a week; ‡ Defined as >2 standard drinks a day, or >4 standard drinks on one occasion a week.

**Table 2:** CR barrier item scores, by assessment point and change.

	1st Administration (pre-CR) (*n* = 186)			2nd Administration (during CR) (*n* = 42)			Mean Change ± SD §	Paired *t*-Test Value for Change §
CRBS Item	Mean Score	SD	Not Applicable *n* (%)	Mean Score	SD	Not Applicable *n* (%)		
01 … of distance	3.08	1.70	7 (3.8%)	1.71	1.33	0	−0.36 ± 0.90	2.23 *
02 … of cost	1.97	1.19	8 (4.3%)	1.32	0.71	1 (2.4%)	−0.22 ± 0.82	1.69
03 … of transportation problems	2.17	1.47	6 (3.2%)	1.12	0.39	1 (2.4%)	−0.27 ± 0.58	2.88 *
04 … of family responsibilities	1.88	1.24	2 (1.1%)	1.29	0.59	0	−0.40 ± 0.82	3.07 *
05 … I didn’t know about CR	1.49	0.92	15 (8.1%)	1.00	0.00	0	−0.38 ± 0.78	3.07 *
06 … I don’t need CR	1.91	1.12	26 (14%)	1.29	0.90	4 (9.5%)	−0.07 ± 0.95	0.48
07 … I already exercise at home or in my community	1.99	1.41	9 (4.8%)	1.41	1.09	1 (2.4%)	−0.14 ± 0.59	1.45
08 … of severe weather	1.79	0.83	108 (58.1%) †	1.20	0.63	1 (2.4%)	−0.05 ± 0.74	0.41
09 … I find exercise tiring or painful	1.62	0.92	10 (5.4%)	1.32	0.77	1 (2.4%)	−0.29 ± 0.96	1.92
10 … of travel	1.96	1.29	11 (5.9%)	1.80	0.96	2 (4.8%)	−0.10 ± 1.08	0.59
11 … of time constraints	2.37	1.55	6 (3.2%)	1.50	0.87	2 (4.8%)	−0.35 ± 1.11	2.00 *
12 … of work responsibilities	2.78	1.39	42 (22.6%)	1.63	1.13	2 (4.8%)	−0.52 ± 1.13	2.88 *
13 … I don’t have the energy	1.79	0.96	10 (5.4%)	1.17	0.53	1 (2.4%)	−0.34 ± 0.83	2.56 *
14 … other health problems prevent me from going	2.13	1.13	65 (34.9%)	1.59	1.13	2 (4.8%)	−0.05 ± 1.37	0.25
15 … I am too old	1.40	0.73	21 (11.3%)	1.03	0.15	4 (9.5%)	−0.22 ± 0.58	2.33 *
16 … my doctor did not feel it was necessary	1.20	0.34	140 (75.3%) †	1.09	0.30	19 (45.2%) †	−0.13 ± 0.75	1.08
17 … many people with heart problems don’t go, and they are fine	1.67	0.98	65 (34.9%)	1.24	0.41	25 (59.5%) †	−0.40 ± 0.84	2.22 *
18 … I can manage on my own	1.90	1.10	51 (27.4%)	1.37	0.88	15 (35.7%)	−0.13 ± 1.16	0.71
19 … I think I was referred but the rehab program didn’t contact me	1.29	0.19	172 (92.5%) †	1.00	0.00	0	−0.33 ± 0.40	4.97 **
20 … it took too long to get referred and into the program	1.38	0.81	31 (16.7%)	1.24	0.61	21 (50.0%) †	−0.25 ± 0.78	2.00 *
21 … I prefer to take care of my health alone	1.54	1.03	31 (16.7%)	1.32	0.90	5 (11.9%)	−0.56 ± 0.94	3.64 **

SD = standard deviation, † = not very relevant (i.e., >50% reported the barrier as not applicable). * *p* < 0.05; ** *p* < 0.001. § In participants who provided data at both assessments.

**Table 3:** Principal components factor analysis results (*n* = 186).

CRBS-CZE Item	Perceived Need §	Logistical Factors †	Work/Time Conflicts ‡	Comorbidities/Health System Factors ∥
17 … many people with heart problems don’t go, and they are fine	0.71 §	0.08	−0.12	−0.02
07 … I already exercise at home or in my community	0.69 §	0.24	0.31	−0.03
06 … I don’t need cardiac rehab	0.65 §	0.08	0.26	0.23
21 … I prefer to take care of my health alone	0.60 §	−0.04	0.06	0.06
18 … I can manage on my own	0.42 §	0.22	0.18	0.06
03 … of transportation problems	−0.04	0.82 †	−0.02	0.09
01 … of distance	0.07	0.78 †	0.05	0.10
02 … of cost	0.17	0.65 †	0.20	0.11
08 … of severe weather	0.11	0.59 †	0.18	−0.11
05 … I didn’t know about cardiac rehab	0.04	0.21 †	0.17	−0.03
11 … of time constraints	0.28	0.17	0.73 ‡	−0.15
12 … of work responsibilities	0.13	0.17	0.66 ‡	−0.22
10 … of travel	0.06	0.03	0.56 ‡	0.07
13 … I don’t have the energy	−0.23	0.03	0.53 ‡	0.37
04 … of family responsibilities	0.19	0.18	0.48 ‡	0.06
14 … other health problems prevent me from going	0.08	0.19	0.05	0.57 ∥
16 … my doctor did not feel it was necessary	0.37	0.14	0.02	0.51 ∥
19 … I think I was referred but the rehab program didn’t contact me	0.35	−0.13	−0.11	0.51 ∥
20 … it took too long to get referred and into the program	0.19	−0.02	−0.13	0.44 ∥
09 … I find exercise tiring or painful	−0.21	0.08	0.40	0.41 ∥
15 … I am too old	0.09	0.26	0.23	0.38 ∥
**Variance explained**	18.85%	9.22%	7.64%	7.15%
**Eigenvalues**	3.96	1.94	1.60	1.50
**Reliability**	0.64	0.69	0.72	0.44

CRBS-CZE = cardiac rehabilitation barriers scale—Czech version. The factor on to which each item loads is shown with the symbols §, †, ‡, ∥.

**Table 4:** Criterion and construct validity of the CRBS-CZE (*n* = 186).

	Perceived Need	Logistical	Work/Time Conflicts	Comorbidities/Health System Factors
**Education**				
University Education (*n* = 34)	1.65 (0.56)	1.62 (0.57)	1.95 (0.62)	1.43 (0.31)
No university (*n* = 152)	1.76 (0.54)	2.05 (0.68) **	2.10 (0.77)	1.63 (0.39) *
**Income**				
Higher income (*n* = 41)	1.72 (0.57)	1.61 (0.57)	2.00 (0.70)	1.47 (0.28)
Lower income (*n* = 145)	1.79 (0.55)	1.96 (0.61) *	2.17 (0.80)	1.63 (0.40) *
**Rurality**				
Urban (*n* = 77)	1.55 (0.51)	1.48 (0.44)	1.79 (0.63)	1.47 (0.37)
Rural (*n* = 109)	1.91 (0.55) **	2.34 (0.63) **	2.29 (0.76) **	1.73 (0.37) **
**AMI**				
Post-AMI (*n* = 99)	1.84 (0.57)	2.06 (0.71)	2.16 (0.76)	1.59 (0.34)
No AMI (*n* = 87)	1.67 (0.51) *	1.86 (0.62) *	1.99 (0.73)	1.60 (0.42)
**Family history of CAD**				
Yes (*n* = 119)	1.59 (0.44)	1.84 (0.56)	1.95 (0.66)	1.48 (0.33)
No (*n* = 87)	1.96 (0.65) **	2.20 (0.81) **	2.30 (0.84) *	1.76 (0.43) **
**Dyslipidemia**				
Yes (*n* = 112)	1.70 (0.56)	1.82 (0.60)	2.02 (0.75)	1.54 (0.41)
No (*n* = 74)	1.80 (0.52)	2.21 (0.73) **	2.18 (0.74)	1.67 (0.33) *
**Use of tobacco**				
Smokers (*n* = 72)	1.77 (0.50)	2.14 (0.74)	2.20 (0.73)	1.64 (0.33)
Non/former smokers (*n* = 114)	1.72 (0.58)	1.87 (0.62) *	2.01 (0.75)	1.56 (0.40)
**Use of alcohol**				
No harmful use (*n* = 154)	1.68 (0.52)	1.96 (0.68)	2.03 (0.72)	1.55 (0.37)
Harmful use (*n* = 32)	1.91 (0.45) *	2.45 (0.89) **	2.34 (0.68) *	1.86 (0.44) **
**Enrollment**				
CR Enrollees (*n* = 45)	1.57 (0.59)	1.53 (0.55)	1.70 (0.58)	1.45 (0.41)
Non-enrollees (*n* = 141)	1.76 (0.52) *	2.16 (0.66) **	2.21 (0.76) **	1.60 (0.36) *
**Completion**				
CR Completers (*n* = 30)	1.28 (0.35)	1.30 (0.38)	1.53 (0.51)	1.31 (0.33)
Non-completers (*n* = 136)	1.86 (0.55) **	2.13 (0.65) **	2.19 (0.74) **	1.69 (0.37) **

SD = standard deviation, AMI = acute myocardial infarction, CR = cardiac rehabilitation; CRBS-CZE = cardiac rehabilitation barriers scale, Czech version; CAD = coronary artery disease. * *p* < 0.05; ** *p* < 0.001. Mean (SD) subscale barrier scores shown.

**Table 5:** Differences in total and subscale CR barrier scores between 1st and 2nd administration of the CRBS-CZE, and by CR completion (*n* = 42).

	1st Administration	2nd Administration		
		Total (*n* = 42)	Completers (*n* = 30)	Non-Completers (*n* = 12)
Total	1.58 (0.47)	1.32 (0.29) *	1.19 (0.15)	1.63 (0.39) ††
Perceived need	1.54 (0.60)	1.27 (0.42) *	1.12 (0.15)	1.67 (0.76) ††
Logistical	1.49 (0.55)	1.27 (0.37) *	1.12 (0.15)	1.63 (0.51) ††
Work/time conflicts	1.69 (0.60)	1.43 (0.44) *	1.29 (0.31)	1.75 (0.56) †
Comorbidities/health system factors	1.42 (0.41)	1.21 (0.27) *	1.15 (0.17)	1.44 (0.42) †

CR = cardiac rehabilitation; CRBS-CZE = cardiac rehabilitation barriers scale, Czech version; SD = standard deviation; * *p* < 0.001 for paired *t*-test comparing CRBS scores from first to second administration. † *p* < 0.05; †† *p* < 0.001 for independent samples *t*-test comparing CRBS scores by CR completion status at second administration. Mean (SD) barriers scores shown.

## Data Availability

The data presented in this study are available on request from the corresponding author. The data are not publicly available.

## Supplementary Materials

The following are available online at https://www.mdpi.com/article/10.3390/ijerph182413113/s1, Table S1: Characteristics of participants completing 2nd CRBS administration.
