# Supplementary material for: Validity and Reliability of the Cardiac Rehabilitation Barriers Scale in the Czech Republic (CRBS-CZE): Determination of Key Barriers in East-Central Europe
Source: Int J Environ Res Public Health. 2021 Dec 12;18(24):13113. doi: 10.3390/ijerph182413113 (PMC8701715; doi:10.3390/ijerph182413113)
Supplement: Supplementary file 1 [file ijerph-18-13113-s001.zip › ijerph-1476627-supplementary.pdf]

Supplementary Table S1. Characteristics of participants completing 2nd CRBS administration.

Characteristics	Total (n = 42)	Completers (*n* = 30)	Non-completers (*n* = 12)	t
**Sociodemographic**				
Age (mean ± SD)	56.8 ± 9.0	57.0 ± 8.8	56.3 ± 9.6	.22
Sex (n, % female)	5 (11.9)	4 (13.3)	1 (8.3)	.45
Marital status ‖ (n, % married)	37 (88.1)	26 (86.7)	11 (91.7)	.45
Educational attainment ‖ (n, % > high school)	21 (50.0)	16 (53.3)	5 (41.7)	.29
Work status ‖ (n, % employed)	35 (83.3)	26 (86.7)	9 (75.0)	.92
Individual income ‖ (n, % > 33,697 CZK/month)	19 (45.2)	13 (43.3)	6 (50.0)	.39
Rurality ‖ (n, % > 30 min commute time to CR one-way by usual mode)	11 (26.2)	5 (16.7)	6 (50.0)	2.37 *
**Clinical**				
Referral indication of CAD ° (n, % yes)	20 (47.6)	17 (56.7)	3 (25.0)	1.93
Post-acute myocardial infarction (n, % yes)	29 (69.0)	21 (70.0)	8 (66.7)	.21
Angina pectoris (n, % yes)	5 (11.9)	3 (10.0)	2 (16.7)	.60
Current/previous PCI (n, % yes)	32 (76.2)	22 (73.3)	9 (75.0)	.11
Current/previous CABG (n, % yes)	2 (4.8)	2 (6.7)	0	.91
Current/previous HF (n, % yes)	2 (4.8)	2 (6.7)	0	.91
Current/previous arrhythmia (n, % yes)	2 (4.8)	2 (6.7)	0	.91
Current/previous valve issue (n, % yes)	3 (7.1)	1 (3.3)	2 (16.7)	1.57
**Risk factors**				
BMI (mean ± SD)	28.9 ± 4.2	29.1 ± 4.6	28.3 ± 2.7	.56
Waist circumference (mean ± SD)	104 ± 12.2	105 ± 12.0	100 ± 12.0	1.21
Family history of CAD ‖ (n, % yes)	26 (61.9)	20 (66.7)	6 (50.0)	1.01
Hypertension ‖ (n, % yes)	26 (61.9)	18 (60)	8 (66.7)	.40
Dyslipidemia ‖ (n, % yes)	31 (73.8)	23 (76.7)	8 (66.7)	.67
Diabetes ‖ (n, % yes)	8 (19.0)	6 (20.0)	2 (16.7)	.24
**Heart-healthy behaviors**				
Physical activity ‖ (n, % inactive §)	17 (40.5)	13 (43.3)	4 (33.3)	.59
Tobacco use ‖ (n, % current)	8 (19.0)	5 (16.7)	3 (25.0)	.62
Use of alcohol ‖ (n, % harmful ‡)	9 (21.4)	4 (13.3)	5 (41.7)	2.13 *
**Psychosocial well-being**				
Stress ‖ (VAS, 1-10; mean ± SD)	5.2 ± 2.5	5.5 ± 2.4	4.4 ± 2.7	1.29
Depression and/or anxiety (n, % yes, diag./self-report ‖)	5 (11.9) / 5 (11.9)	4 (13.3) / 4 (13.3)	1 (8.3) / 1 (8.3)	.45
**CR utilization**				
Adherence (mean % of sessions completed ± SD)	75.8 ± 24.9%	89.1 ± 7.4%	41.9 ± 21.5%	10.69 **

SD = standard deviation, CRBS = Cardiac Rehabilitation Barriers Scale, CR = cardiac rehabilitation, CAD = coronary artery disease, PCI = percutaneous coronary intervention, CABG = coronary artery bypass graft, HF = heart failure, BMI = body mass index, VAS = visual analogue scale; ‖ Presents self-report data. All other data measured or extracted from patient charts; * $p < 0.05$; ** $p < 0.001$. ° Defined as coronary artery stenosis > 50%, primomanifestation. § Defined as > 150/75 mins of moderate/vigorous intensity physical activity a week; ‡ Defined as > 2 standard drinks a day, or > 4 standard drinks on one occasion a week.
